# The value of high-frequency and color Doppler ultrasonography in diagnosing congenital muscular torticollis

**DOI:** 10.1186/1471-2474-13-209

**Published:** 2012-10-26

**Authors:** Lei Wang, Lingyan Zhang, Yuanjiao Tang, Li Qiu

**Affiliations:** 1 Department of Ultrasound, West China Hospital of Sichuan University, No.37 Guo Xue Xiang, Chengdu, Sichuan Province, 610041, China

**Keywords:** Sternocleidomastoid muscle, Congenital muscular torticollis, Musculoskeletal imaging, High-frequency ultrasonography, Color Doppler ultrasonography

## Abstract

**Background:**

Congenital muscular torticollis (CMT) is a relatively common neck deformity in infancy. The aim of our research was to determine the value of high-frequency and color Doppler ultrasonography in diagnosing CMT.

**Methods:**

Patients with a clinical suspicion of CMT underwent an ultrasound examination before diagnosis, and the sonographic characteristics were analyzed and compared with the clinical findings.

**Results:**

The sensitivity and specificity of an ultrasound diagnosis for CMT was 95.83% and 83.33%, respectively. The patients were divided into 2 groups based on the stage of the disease: the early-stage group (age <1 year) and the late-stage group(age ≥1 year). Differences existed between the two groups with respect to sonographic findings and clinical characteristics. The sonographic characteristics of the early-stage group included local thickening of the sternocleidomastoid muscle (SCM), weak or uneven echoes, and blood flow signals around or inside most of the lesions. The sonographic characteristics of the late-stage group included diffusely hyperechoic, or cord-like hyperechoic signals inside the muscle layer without significant blood flow signals.

**Conclusions:**

Different stage of CMT patients had different sonographic characteristics. High-frequency and color Doppler ultrasonography can serve as adjunct confirmation tool for the diagnosis of CMT.

## Background

Congenital muscular torticollis (CMT) is a relatively common neck deformity in infancy, with a reported incidence of 0.3% - 1.9% [1]. The lesion is typically located in the sternocleidomastoid muscle (SCM). No deformity is apparent at birth, but a neck mass appears 7–10 days after birth, and resolves several months later, after changing into contractures and fibrosis of the SCM. This results in an inclination of the head toward the affected side. Without treatment, facial and head deformities develop over time, which may seriously affect the patient’s work and quality of life [1]. The direct causes of SCM fibrosis are excessive deposition of extracellular matrix collagen and changes of other extracellular matrix components [2].

Self-healing is possible in patients with CMT <1 year of age (early stage), and conservative treatment has yielded satisfactory outcomes. Therefore, most researchers have suggested that surgery be performed only when the patient is > 1 year of age (late stage) [3]. In the current study, we analyzed the accuracy of a SCM ultrasound examination and sonographic characteristics during the early and late stages in patients with CMT who were treated in our hospital between 2008 and 2010, and determined the diagnostic value of high-frequency and color Doppler ultrasonography, as well as the imaging features of CMT.

## Methods

### Subjects

The present study involved 108 patients with wryneck or neck masses who were suspected to have CMT and were treated in our hospital between January 2008 and December 2010. Study approval was waived by the Institutional Review Board (IRB) as this was considered a diagnostic procedure for patient benefit. Informed consent was obtained by the parents of the affected children. Forty and 68 patients sought evaluation at our hospital for neck masses and wryneck, respectively. Ninety-six of the 108 patients were diagnosed with CMT after surgical or other clinical treatments. The surgical diagnosis of CMT was based on pathologic changes (stromal proliferation and fibrosis) in the affected SCM. The clinical diagnosis was based on the neck mass or wryneck resolving after rehabilitative treatment for CMT. In the patients who do not respond to conservative treatment surgical release of the muscle was done. The US assesment of CMT performed after the initial clinical assessment and before the start of any treatment (rehabilitation and surgery) for CMT. Twelve patients were diagnosed with non-muscular torticollis including bony, ocular, and spastic torticollis. Of the 96 patients with CMT, 56 were male and 40 were female. The mean age of the patients was 17.9 ± 18.2 months, with a range of 15 days to 5 years. The duration of disease ranged from 1 month to 3 years. Forty-six of the 96 patients underwent surgery, and 50 patients received rehabilitative treatment. Of the 46 patients who underwent surgery, 26 were male and 18 were female, and the mean age was 33.7 ± 14.0 months (range, 10 months to 5 years); all of the patients had wryneck. Of the 50 patients who received rehabilitative treatment, 30 were male and 22 were female, and the mean age was 3.3 ± 2.9 months (range, 15 days to 15 months); 40 and 22 patients sought evaluation for neck masses and wryneck, respectively.

### Ultrasonography

Ultrasound examination were performed on all patients using Philips HD-11 and IU22 ultrasound instrument (Philips Healthcare, Andover, MA, USA). The frequency of the probe was 8–12 MHz. Gray-scale and power Doppler ultrasound examinations were performed using standardized examination techniques, standardized definitions of ultrasound terms [4], and standardized power Doppler ultrasound settings (pulse repetition frequency, 750 Hz; and color gain just below the background noise level). The musculoskeletal low-speed blood flow condition was selected. The ultrasound equipment was operated by an experienced sonographer who had been performing musculoskeletal ultrasonograms for 8 years. The sonographer did not know the clinical diagnoses of the patients during the examination. To avoid patient movement during the examination, sonography was performed while sleep. The head was rotated to the opposite side, and longitudinal and transverse scanning was performed directly on the lateral side of the neck, focusing on the SCM. Abnormal findings were documented in two perpendicular planes (longitudinal and transverse) to determine whether or not there were any changes in the thickness or intramuscular texture of the SCM and whether there were abnormal echo signals. A bilateral examination was performed The thickness of the SCM was measured twice at the thickest location to obtain an average value in the transverse section. A color Doppler ultrasonographic examination was performed to determine whether or not there were blood flow signals inside the lesion.

### Data analysis

SPSS 17.0 (SPSS, Inc., Chicago, IL, USA) was used to evaluate the data. All measurement data were represented as the mean ± standard deviations. Different descriptive statistics were used depending on the normality assumption (Kolmogorov-Smirnoff test). A diagnostic test fourfold table was used to compare the results of the ultrasound examination and the results of the final diagnosis by surgery or clinical treatment follow-up. The chi-squared test was used to compare the ultrasound characteristics during early-and late- stage CMT. A two tailed *P* < 0.05 was considered statistically significant.

## Results

### Ultrasound diagnostic test of CMT

Ninety-six of 108 patients were diagnosed with CMT, and 92 patients had an abnormal SCM on ultrasonographic examination; no SCM abnormalities were demonstrated in the other 4 patients. Of the 12 patients who were initially diagnosed with bony, ocular, or spastic non-muscular torticollis, 10 were shown to have no abnormalities in SCM thickness or echo signals. The remaining two patients had slightly thickened SCMs, but no abnormal echo signal findings. The surgical or clinical therapy and follow-up results are considered to be the gold standard for CMT diagnosis, with ultrasound examination considered a diagnostic test; a four-fold table was established (Table 1). The sensitivity and specificity of ultrasound in diagnosing CMT was 95.83%, and 83.33%, respectively. The positive likelihood ratio was 5.75 and the negative likelihood ratio was 0.05.

**Table 1:** Ultrasound diagnostic test of congenital muscular torticollis (CMT)

		**Patients with CMT**	**Patients without CMT**	**Total**
Ultrasound	+	92	2	94
	-	4	10	14
Total		96	12	108

The sensitivity of ultrasound in diagnosing CMT was 95.83%, and the specificity was 83.33%.

### Comparison between clinical characteristics and ultrasound features in early- and late- stage CMT

The ultrasound examinations revealed abnormal SCM thicknesses and echo signals in 92 patients initially diagnosed with CMT. These patients were divided into early- and late-stage groups based on patient’ age. Fifty-six patients (60.87%) were in the early-stage group (age <1 year) and 36 patients (39.13%) were in the late-stage group (age ≥1 year). The lesion was located on the left side in 52 cases (56.52%) and the right side in 40 cases (43.49%). The comparison of clinical and ultrasonographic features in the early and late stages showed that all lesions were located at the middle and lower segments of the SCM, with no significant difference observed between the two groups. However, statistically significant differences existed between the two groups with respect to other ultrasound features, including lesion boundaries, SCM thicknesses, echo signals from the lesion, color Doppler flow imaging, and clinical characteristics, such as the presence of palpable neck masses, neck activity, and treatment methods (*P* < 0.05) (Table 2).

**Table 2:** Comparison of ultrasonographic features and clinical characteristics in the early and late stages of congenital muscular torticollis (CMT)

		**Ultrasound diagnosis of CMT**		
		**Early stage (56 cases)**	**Late stage (36 cases)**	***P*value**
	Location of SCM lesion	At middle and lower segments in all 56 cases	At middle and lower segments in 34 cases, and at upper and lower segments in 2 cases (dumbbell-shaped)	>0.05
	Boundary of SCM lesion	Clear in 52 cases and not clear in 4 cases	Diffuse without marked boundaries in 20 cases, clear in 16 cases	<0.05
Ultrasound features	Change in SCM thickness	Thickening in all 56 cases	Thickening in 16 cases, thinning in 20 cases	<0.05
	Echo signals from the affected SCM	Weak in 40 cases, uneven in 16 cases	Diffuse increase in 16 cases, cord-like hyperechoic signals in 16 cases, decrease in 4 cases	<0.05
	Blood supply in color Doppler	Spotty and cord-like blood flow signals in 36 cases, no marked blood flow signals in 20 cases	No marked blood flow signals in all 36 cases	<0.05
	Neck mass	Significant in 52 cases, not apparent in 4 cases	Neck masses in 8 cases, no neck masses in 28 cases	<0.05
Clinical characteristics	Neck activity	Mild limitation in lateral bending to the affected side in 26 cases, marked rotational limitation in 8 cases, no marked limitation in 22 cases	Rotational limitation in 34 cases, mild limitation in lateral bending to the affected side in 2 cases	<0.05
	Treatment	Rehabilitation in 48 cases, surgery in 8 cases	Surgery in 34 cases, rehabilitation in 2 cases	<0.05
	Pathology	Stromal proliferation and fibrosis in SCM in 8 cases	Stromal proliferation and fibrosis in SCM in 34 cases	<0.05

**Figure 1:**
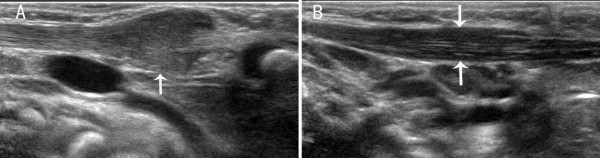
**A. A vertical section of the sternocleidomastoid muscle (SCM) in a patient with early-stage muscular torticollis. B**. Vertical section of the SCM of the normal side. Thickening of the affected SCM is apparent, and there are hypoechoic signals without a clear boundary. The boundary between muscular fibers is not clear, with a disappearance of the inner muscle texture.

**Figure 2:**
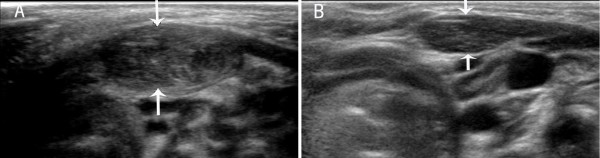
**A. Cross-section of the sternocleidomastoid muscle (SCM) in a patient with early-stage muscular torticollis. B**. Cross-section of the SCM of the normal side. Thickening of the affected SCM is apparent, and there are hypoechoic signals with a clear boundary and disappearance of the inner muscle texture.

**Figure 3:**
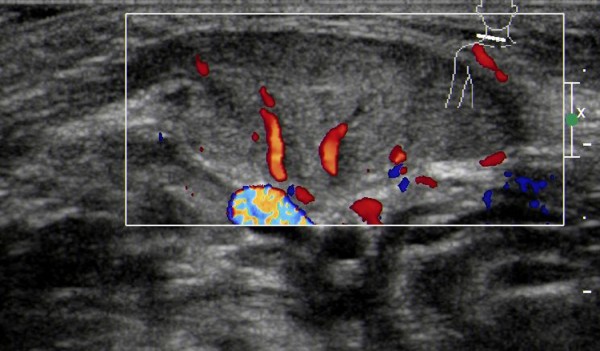
**Thickening of the sternocleidomastoid muscle in a patient with early-stage muscular torticollis, with hypoechoic signals and cord-like blood flow signals**.

**Figure 4:**
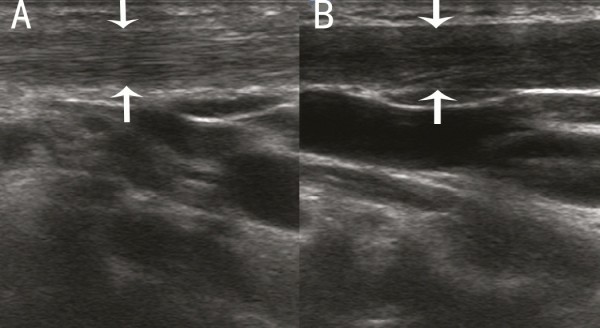
**A. Vertical section of the sternocleidomastoid muscle (SCM) in a patient with late-stage muscular torticollis. B**. Vertical section of the SCM of the normal side. The affected sternocleidomastoid muscle is thinned, and diffuse hyperechoic signals are apparent.

**Figure 5:**
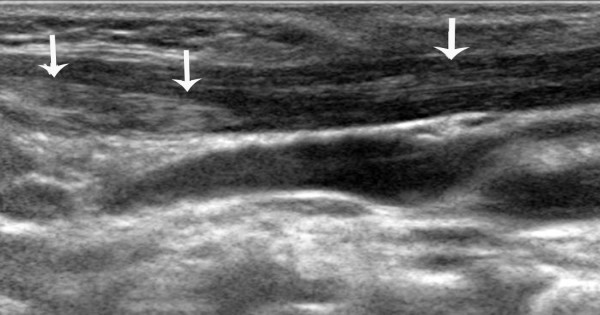
**Vertical section of the sternocleidomastoid muscle (SCM) in a patient with late-stage muscular torticollis.** Cord-like hyperechoic signals are apparent inside the affected SCM, with a clear boundary.

**Figure 6:**
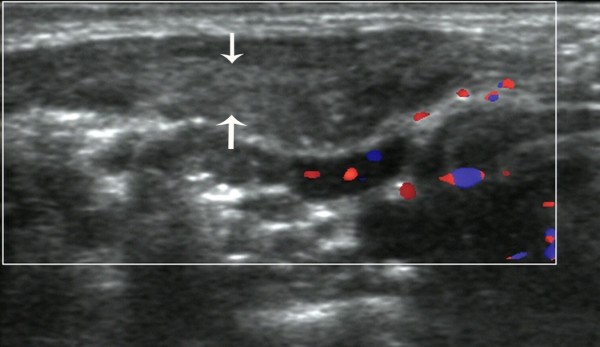
**Cross-section of the sternocleidomastoid muscle (SCM) in a patient with late-stage muscular torticollis.** Hyperechoic signals are apparent inside the affected SCM, and no significant blood flow signal is seen.

The sonographic features of patients in the early stage included local thickening of the affected SCM, weak or uneven echoes, and a relatively clear boundary (the boundary between SCM muscle fibers was clear in the cross-section, but not clear in the vertical section; Figures 1 and 2). Color Doppler ultrasonography showed short rod- or cord-like blood flow signals inside or around the thickened SCM in some cases (Figure 3), and spectra of arterial and venous flow were measurable. In the late stage, the affected SCM was thick or thin, and there were diffuse increases in echo signals (Figure 4) or cord-like hyperechoic signals inside the muscle layer (Figure 5). The boundary was not clear when there was a diffuse increase, and clear when there were cord-like hyperechoic signals; no significant blood flow signal existed inside the affected SCM on color Doppler (Figure 6). The clinical data showed that the main clinical manifestation in the early stage was a neck mass, which resolved during the late stage. Neck activity was more limited in the late stage than the early stage. Rehabilitation was carried out mostly for the early-stage group, and surgery was mostly performed for the late-stage group.

## Discussion

CMT is a relatively common deformity amongst infants.The direct cause of CMT is contracture and shortening of the SCM as a result of muscle fibrosis;however, the reason for muscle fibrosis is unclear [5]. Recently, it has been found that the extent of SCM fibrosis is closely related to a limitation in the birth canal and an abnormal fetal position [6]. The basic pathologic changes in patients with CMT are stromal proliferation and SCM fibrosis. Electron microscopic observations have shownthat the proliferated stroma contains collagen fibers and fibroblasts, together with large numbers of myoblasts, myofibroblasts, and mesenchymal-like cells. Some researchers believe that the key factor underlying different clinical outcomes of neck masses is the ratio of fibroblasts to myoblasts at various phases of differentiation and degeneration [7]. The mass will disappear if myoblasts inside the mass differentiate and develop, as during the embryonic period. If degeneration takes place in some parts of myoblasts, partial muscle contracture occurs. If degeneration occurs in the majority of myoblasts, fibroblasts produce large amounts of collagen, leading to scar-like contracture of the SCM and typically resulting in torticollis [8]. The timing of CMT treatment in the recovery of muscle function is key to the outcome; the sooner treatment begins, the better the outcome is likely to be. Self-healing of CMT is possible in patients < 1 year of age, and conservative treatment may achieve satisfactory results at this stage. Therefore, most researchers suggest only performing surgery in patients >1 year of age [9]. If a diagnosis of CMT is established early and conservative treatment is initiated quickly, the cure rate can be > 80%, and the prognosis is excellent [9]. If contractures or local deformities of the SCM have already occurred, the prognosis is poor, even after surgical treatment [10]. Ultrasound examinations can reveal abnormal thicknesses and echo signals in the SCM in patients with CMT during the early stage. Our study showed that as a diagnostic test for CMT, ultrasonography has a very high sensitivity and specificity, which makes ultrasonography a very effective diagnostic tool. In our study, ultrasound examinations revealed no abnormalities in four patients who were diagnosed with CMT after surgery. Surgery showed that fibrosis occurred at the insertion sites of the sternal and clavicular heads of the SCM. It is possible that the sonographer neglected to check the distal ends of the SCM during scanning, resulting in misdiagnosis. In patients with non-muscular torticollis, mild SCM thickening occurred in two cases without abnormal echo signals, and the patients were ultimately diagnosed with spastic torticollis; however, there were no abnormal echo signal inside the muscle. Therefore, the diagnosis of CMT should not be based only on change in SCM thickness, but changes in echo signals should also be taken into account.

We also demonstrated differences in the clinical characteristics of CMT between the early and the late stages. The major manifestation in the early stage is a neck mass, while the characteristics in the late stage include muscle tension, contractures, and reduced neck activity. The stage of the disease affects the selection of treatment methods, with rehabilitative therapy often selected in the early stage and surgical treatment in the late stage.

Most of the previous studies have reported that the following sonographic features of the affected SCM in patients with CMT: echogenicity; texture; motility; softness; and the transverse and longitudinal extent of the involvement [11-14]. Although many reports have been published on the ultrasonographic features of CMT, studies differentiating such features between the early and late stages are rare. Because the clinical manifestations of the disease, the extent of fibrosis, and treatment methods differ by stage, differences in ultrasound features in the early and late stages are valuable to help assess the severity of the disease and select an appropriate treatment method. We found that sonographic features of patients in the early stage of CMT include local thickening of the SCM, and weak or uneven echoes. In the late stage of CMT, the affected SCM becomes thick or thin, and there are diffuse increases in echo signals or cord-like hyperechoic signals inside the muscle layer. Color Doppler ultrasonography revealed short rod- and cord-like blood flow signals inside the thickened SCM muscle in patients with early-stage CMT (age <1 year). This finding may be associated with the rich network of capillaries inside the reactive granulation tissue that is produced during delivery because of extension of the SCM muscle fibers under heavy loads from external forces and gravity [15]. No significant blood flow signal was noted inside the affected SCM in patients with late-stage CMT (age ≥1 year),which may be associated with the organization of granulation tissue, increased fibrosis, and reduced number of blood vessels. The results of the current study suggest that the color Doppler presentation can help determine the stage of the disease and the extent of fibrosis [16].

Some researchers have indicated that if there is persistent head tilting and notable neck inclination to the affected side (>15°), as well as muscle contractures or scleromas inside the muscle after 6 months of conservative treatment, surgery should be performed early [17]. In patients with CMT and ultrasonographic manifestations of the late stage, such as diffuse increases in echo signals or cord-like hyperechoic signals inside the muscle layer which meant relatively severe fibrosis, surgical treatment is used in most cases.In agreement with Yu *et al.*[18], for patients with facial deformities caused by CMT, we believe that early surgery should be performed, even at age ≤ 8 months of age. If fibrosis is seen in a patient > 1 year of age, surgery should be performed as soon as possible to prevent refractory facial and neck deformities. In the present study, ultrasound examinations showed diffuse hyperechoic signals of the SCM and notable neck inclination in 8 cases during the early stage. Surgical treatment was performed in these patients, and significant muscular fibrosis was found intra-operatively. This suggests that when an ultrasound examination reveals diffusely increased or cord-like hyperechoic signals inside the SCM lesion and no blood flow signal within the lesion, indicating significant SCM fibrosis, surgery rather than rehabilitative therapy should be offered, even if the patient is < 1 year of age. Thus, an ultrasound examination can guide treatment decisions for patients with CMT [19].

Non-muscular torticollis may be life-threatening if not treated early. In the present study, the SCM echo signals were normal in patients with non-muscular torticollis, with the exception of one case with a slightly thickened SCM. Muscular torticollis and non-muscular torticollis can be differentiated by the combination of changes in SCM thickness and echo signals. An ultrasound examination can differentiate a mass from other types of masses, such as cervical cystic lymphangiomas and enlarged cervical lymph nodes, and thus has high clinical value [20].

## Conclusions

To conclude, CMT patients at different stages of disease have different sonographic characteristics. Ultrasound examinations can serve as adjunct confirmation tools for the diagnosis of CMT.

## Abbreviations

CMT: Congenital muscular torticollis; SCM: Sternocleidomastoid muscle.

## Competing interests

The authors declare that they have no competing interests.

## Authors’ contributions

LW: Writing the paper; LYZ: Supervision of the study and the final version of the paper; YJT: Echographic evaluation; LQ: Planning of the study. All authors read and approved the final manuscript.

## Funding

This work was supported by grants from National Natural Science Foundation of China (approval number: 81271585). No additional external funding received for this study.

## Pre-publication history

The pre-publication history for this paper can be accessed here:

http://www.biomedcentral.com/1471-2474/13/209/prepub
